# A Behaviorally-Explicit Approach for Delivering Vaccine Baits to Mesopredators to Control Epizootics in Fragmented Landscapes

**DOI:** 10.1371/journal.pone.0113206

**Published:** 2015-01-14

**Authors:** James C. Beasley, Todd C. Atwood, Michael E. Byrne, Kurt C. Vercauteren, Shylo R. Johnson, Olin E. Rhodes Jr.

**Affiliations:** 1 University of Georgia, Savannah River Ecology Laboratory, Aiken, South Carolina, United States of America; 2 USDA APHIS Wildlife Services, National Wildlife Research Center, Fort Collins, Colorado, United States of America; Thomas Jefferson University, UNITED STATES

## Abstract

Despite the widespread use of aerial baiting to manage epizootics among free-ranging populations, particularly in rabies management, bait acceptance and seroconversion rates often are lower than required to eliminate spread of disease. Our objectives in this study, therefore, were to evaluate the performance of stratified bait distribution models derived from resource selection functions (RSF) on uptake of placebo rabies baits by raccoons (*Procyon lotor*) and Virginia opossums (*Didelphis virginiana*), as well as the probability of bait uptake as a function of proximity to bait distribution areas in fragmented agricultural ecosystems. Among 478 raccoons and 108 opossums evaluated for presence of Rhodamine B (RB) across 8 sites, only 26% of raccoons and 20% of opossums exhibited marking consistent with bait consumption 14–24 days post-baiting. The effective area treated, based on 90% kernel density estimators of marked individuals, ranged from 99–240 ha larger than bait distribution zones, with RB marked individuals captured up to 753m beyond the bait zone. Despite incorporation of RSF data into bait distribution models, no differences in uptake rates were observed between treatment and control sites. These data likely reflect the underlying constraints imposed by the loss and fragmentation of habitat on animal movement in heterogeneous landscapes, forcing individuals to optimize movements at coarse (i.e., patch-level) rather than fine spatial scales in highly fragmented environments. Our data also confirm that the probability of bait acceptance decreases with increasing distance from bait zone interiors, even within the zone itself. Thus, although bait acceptance was confirmed beyond bait zone boundaries, the proportion of vaccinated individuals may comprise a small minority of the population at increasing distances from baiting interiors. These data suggest focal baiting creates a buffered area of treated individuals around bait zones or bait stations, but repeated treatments may be needed to achieve sufficient uptake to eradicate disease.

## Introduction

Over the last few decades, the use of baits to deliver pharmaceuticals (vaccines, fertility control, toxicants) has emerged as one of the most widely applied tools in the management and conservation of wildlife species across the globe. In particular, aerial dissemination of baits has become the primary means of distributing vaccines across large spatial scales to manage epizootics (e.g., rabies) among free-ranging wildlife populations [1, 2]. For example, intensive oral vaccination programs have been established throughout many areas where rabies is endemic as part of ongoing attempts to eliminate this disease from wildlife reservoirs [1, 3–5]. These efforts, which rely on the uptake of an oral vaccine enclosed in a palatable bait matrix or sachet, have successfully eliminated rabies from free-ranging populations of red fox (*Vulpes vulpes*) in several European countries [6] and a canine rabies virus variant from coyotes (*Canis latrans*) in south Texas, USA [7–8]. Similarly, baiting programs have been established to control the spread of numerous parasites, including *Baylisascaris procyonis* in Allegheny woodrats (*Neotoma magister*) [9–11] and *Echinococcus multilocularis* in red fox (*Vulpes vulpes*) [12–14]. Baiting programs to vaccinate for *Mycobacterium bovis* in Eurasian badgers (*Meles meles*) also are currently being developed [15–16].

Although oral vaccination programs have successfully eliminated or reduced the spread of zoonoses from some regions [1, 6–8], eradication of rabies from terrestrial wildlife reservoirs represents one of the greatest challenges among current wildlife disease programs. One of the primary challenges stems from the fact that a diverse assemblage of mesocarnivore species serve as reservoirs, each for specific variants of the virus. These species range considerably in size, diet, and life history, and as a result universal bait types or baiting strategies, although logistically tractable, may not be optimal [17]. Complicating this issue, bait acceptance and the proportion of populations exhibiting immunity following baiting regimes vary geographically among species [18–19], and thus a multi-dimensional approach tailored to individual species or regional conditions may be needed to combat the spread of this disease.

In North America raccoons (*Procyon lotor*) represent >50% of confirmed rabies cases in terrestrial wildlife species and thus the majority of baiting and management efforts have targeted this species [1, 20]. Much effort has focused on distributing vaccine-laden baits along a north-south corridor ranging from Maine to Florida to prevent the spread of raccoon-variant rabies westward [1, 17]. Although these efforts have essentially halted the expansion of raccoon-variant rabies, bait acceptance and seroconversion rates for raccoons remain between 30% and 40% in many regions [1, 9]; but see [18], far from the ~70% believed necessary to eliminate the disease in free-ranging populations [21–23]. Factors such as inadequate baiting density, non-target bait consumption, bait type (palatability), bait distribution patterns, and timing of vaccine distributions all have been identified as potential factors contributing to sub-optimal vaccination rates of free-ranging populations [17, 24–26], yet clear solutions to integrate site-specific and regional influences of these and other variables are often lacking (but see [27]). Additional research is needed to elucidate the effects of these and other potential confounding variables, particularly for areas beyond the existing ORV barrier, to aid in the continued refinement of ORV protocols.

The composition and configuration of habitats directly influence the movement behavior and population dynamics of wildlife, particularly in heterogeneous landscapes [28–32]. Yet, despite the influence of habitat on animal movement behavior, current programs distributing pharmaceutical baits rarely incorporate habitat attributes into bait distribution strategies [25, 33]. The development of bait distribution strategies that rely on resource selection data to guide stratification of bait densities should maximize bait encounter rates and the effective area baited for target species, potentially improving vaccination rates and reducing competition with non-target species. Indeed, Vos et al. [34] observed higher bait disappearance rates in forested habitats compared to agricultural areas, suggesting that stratifying bait density relative to patterns of resource selection may increase bait encounter rates as well as the effective area baited; although that study did not quantify species responsible for bait removal. This could be particularly important in the heavily fragmented landscapes of the midwestern U.S., where the advancement of raccoon rabies could create new and unique challenges to containing this disease. Agricultural ecosystems support elevated densities of raccoons and individuals exhibit unique movement patterns, which could be manifested as temporary movement in and out of potential bait treatment areas, due to the highly fragmented distribution of native habitats and seasonal availability of crops [30, 35–36]. Moreover, Virginia opossums (*Didelphis virginiana*), which do not serve as reservoirs of rabies, also occur at high densities in agricultural ecosystems and are important bait competitors that can reduce bait availability to raccoons [9].

The objectives of this research, therefore, were to 1) quantify uptake of placebo rabies baits by both raccoons and Virginia opossums, 2) evaluate the performance of stratified bait distribution models based on predicted space use patterns derived from resource selection models, and 3) quantify the effective area treated following localized bait distributions as well as the probability of bait consumption as a function of proximity to bait distribution areas. Given the limited use of crop-field interiors by raccoons in agricultural ecosystems [37], we limited areas selected for this research to forest tracts >~100 ha as our primary interest was in determining whether incorporation of fine-scale habitat attributes into bait distribution models significantly alters uptake rates by raccoons. We hypothesized that incorporation of resource selection models into bait distribution patterns would increase bait acceptance rates by raccoons and that effective baiting areas would exceed bait distribution zones, but that the probability of bait consumption would decrease as a function of distance from bait distribution centers.

## Materials and Methods

### Ethics Statement

All trapping and handling methods conformed to the American Society of Mammalogists guidelines [38] as well as Purdue University Animal Care and Use Committee policies under Protocol 01–079. Collection permits for this research were obtained from the Indiana Department of Natural Resources.

### Study Area

This study was carried out in portions of the Upper Wabash River Basin (UWB) in north-central Indiana, USA. Although originally dominated by deciduous hardwood forests, this landscape underwent extensive deforestation subsequent to European colonization. At the time of this study, approximately 96% of the land area was privately owned and 88% of the UWB was in agricultural production, primarily of corn and soybeans. Only 8% of the basin was forested, with forested habitats dominated by oak (*Quercus*), hickory (*Carya*), and maple (*Acer*). Due to the intensive agricultural activity, forest habitat in the UWB was highly fragmented, with the majority of larger forest tracts confined to major drainages where frequent flooding or locally steep topography made the land unsuitable for agricultural production.

**Figure 1:**
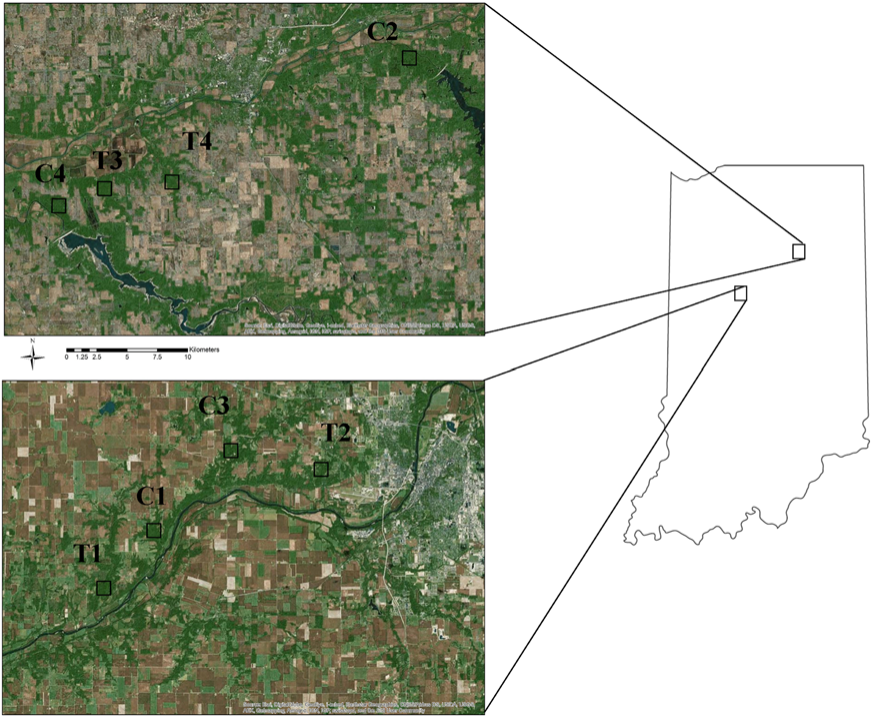
Map of study area showing the distribution of control ‘C’ and treatment ‘T’ sites throughout the Upper Wabash River Basin in Indiana, USA. Boxes represent actual bait distribution zones within each study site.

Within agricultural ecosystems, raccoon activity is primarily concentrated within forested habitat or along forest-agricultural interfaces, with little activity occurring within the agricultural matrix [31, 35–37]. Thus, we selected 8 study cells throughout the UWB that were primarily comprised of large tracts of forested habitat to carry out this study. Given the limited use of crop field interiors by raccoons in agricultural ecosystems, small forest patches surrounded primarily by agriculture were excluded as our primary objective was to evaluate the performance of fine-scale resource selection models within forested areas. Four of the study cells were randomly assigned as treatment sites [T1-T4, bait density determined via Resource Selection Function (RSF)] and the remaining 4 as controls (C1-C4, bait density uniform; Fig. 1). Each cell was approximately 5km^2^ in size and within the center of each cell we established a 73 ha bait distribution zone based on the average home range size of raccoons in our study area (73 ha; [39]). A minimum linear distance of 2 km was maintained between bait distribution zones to minimize chances of between-site movements as this distance exceeds the largest home range observed for raccoons in our study area [39].

### Initial Mark-Recapture Sampling

During March-April 2011, we trapped raccoons in each of the 8 study cells to obtain a baseline estimate of raccoon density and determine the appropriate number of baits to distribute within each cell. Trapping was restricted to within the 73 ha bait distribution zone where we established 2 trapping grids separated by approximately the radius of an average raccoon home range in our study area ($\bar{X}=542$ m; [39]). Each trapping grid consisted of 30 box live traps (Tomahawk Live Trap Co., Tomahawk, Wisconsin, USA) baited with commercial cat food and spaced 50m apart [30]. Traps were pre-baited for 3 nights and were subsequently opened and checked for 10 consecutive nights. We immobilized captured raccoons and Virginia opossums with Telazol (Fort Dodge Animal Health, Fort Dodge, IA, USA) at a dosage of 5 mg/kg of estimated body mass. We ear-tagged (Monel #3, National Band and Tag Company, Newport, Kentucky, USA) all captured individuals and collected standard morphological and demographic data. Raccoons were classified as juveniles (1 yr), yearlings (2 yr), or adults (≥3 yr) and opossums were distinguished as juveniles (~1 yr) or adults (≥2 yr) based on tooth wear.

To estimate raccoon abundance for determination of bait distribution numbers, we used the Huggins closed population modeling procedure in Program MARK [40]. For some trapping grids we captured too few individuals (range: 6–29, $\bar{X}=13.6$) to accurately estimate patch-specific detection probabilities. To overcome this issue we pooled capture histories for all individuals captured at a site (i.e. both trapping grids combined) to derive robust estimates of capture (*p*) and recapture (*c*) parameters. Although combining capture histories assumes equal capture and recapture probabilities exist between grids, we believe this is a realistic assumption given the proximity of the concurrently trapped grids to one another. We developed separate models for each study site and incorporated both sex and age of raccoons as covariates. We evaluated model fit using a bias-corrected version of Akaike’s Information Criterion adjusted for small sample sizes (AIC_c_) and used model averaging to determine final population sizes for all models deviating ≤2 AIC_c_ units from the top model [41].

Based on MARK estimates of population sizes, we estimated raccoon densities within each site by dividing population size by the effective trapping area. We determined effective trapping areas by overlaying a buffer encompassing an area equal to the average home range size of raccoons in our study area (73 ha; [39]), centered on the trapping grid to account for raccoon movement beyond the trapping grid boundaries. For each site we then overlaid and merged trapping grid buffers in ArcGIS (Environmental Systems Research Institute, Inc., Redlands, CA) to approximate the total effective trapping area (km^2^) of trapping grids for each site.

### Bait Distribution

In both control and treatment sites baits were distributed within 3 days following the conclusion of the initial 10-day trapping period along 6 pre-defined transects throughout each 73 ha bait zone. Bait zones were square in shape and we used GIS to establish six 865 m transects spaced 150 m apart in a North-South orientation within each zone. In the field transects were located and traversed using a handheld GPS. Placebo baits consisted of 150 mg of Rhodamine B (RB) incorporated into a fishmeal polymer matrix (Bait-Tek Inc., Orange, TX, USA). These baits (sans RB) were identical to fishmeal polymer baits distributed by the USDA ORV program, with the exception that no vaccine was included.

At control sites, baits were distributed uniformly along the 6 transects at a density of 10 baits per raccoon, based on abundance estimates derived from initial mark-recapture sampling. Bait densities for each study area were estimated as: density estimate derived for each site (see above)*area of the bait zone*10 (i.e. baits/raccoon). Thus, due to differences in raccoon abundance, actual densities of baits distributed ranged from 69 to 162 baits/km^2^ ($\bar{X}=126$) within control sites. Uniform distances between baits varied among control sites and were shorter for those sites with higher raccoon abundance due to the higher number of baits per km^2^ required.

Within treatment sites baits were again distributed along 6 equidistant transects within each bait zone based on local population estimates of raccoons, but were distributed in a stratified, rather than uniform, design based on observed patterns of resource selection for raccoons in our study area. As with control sites, the number of raccoons used for determination of bait distributions was based on population estimates derived from our MARK models, assuming that raccoon densities were uniform throughout each 73ha study site. To stratify bait zones based on raccoon resource selection patterns, we incorporated a RSF developed by Beasley et al. [42]. Briefly, RSF’s are a statistical representation of the habitat associations of organisms that can be used in a probabilistic framework to characterize the distribution of animal movements within a landscape. This RSF was derived from 1,162 locations collected for 17 individuals (7 male, 10 female) fitted with VHF transmitters and tracked from September 2009 to May 2010 within our study area. From these locations fixed kernel 95% and 25% utilization distributions (UD’s—i.e. probability distribution representing the frequency of space use for organisms within a landscape) were generated for each individual and used to estimate the RSF based on distance metrics for forest, agriculture, water, developed, and shrubland habitat attributes within 25% UD’s compared to those of the surrounding landscape (See [42] for additional RSF estimation details). The resulting RSF equation was: *RSF* = exp(0.6473 + 0.0041(distance to agriculture) − 0.023(distance to forest) − 0.0034(distance to water))

We then fit our RSF to each of 5,000 random points generated within each treatment zone using ArcGIS. Each point was ranked and classified as low, moderate, or high probability of use based on the lower, middle, and upper third of the distribution of RSF scores. Points of the same classification (e.g., low) were then collated to generate unique polygons for each selection category in ArcGIS. Baits were distributed along transects based on their intersection with these polygons; portions of each transect that fell within low, moderate, and high quality habitat polygons were provisioned with densities of 5, 10, and 15 baits per raccoon, respectively (see above for conversion from baits/raccoon to bait density). For example, if the first half of a transect was contained within a single polygon of “low” quality habitat and the remaining half of that transect was within a single polygon of “medium” quality habitat, baits would be distributed at a density of 5 and 10 baits/raccoon, respectively within each half of the transect. Polygon boundaries were incorporated into GPS units to facilitate bait distribution. Actual densities of baits distributed within treatment sites ranged from 126 to 152 baits/km^2^ ($\bar{X}=134$).

### Post-Baiting Mark-Recapture Sampling

Two weeks subsequent to the distribution of RB baits, we re-trapped each study cell to collect whiskers for determination of bait uptake patterns [9]. Traps were spaced 75 m apart in a web design radiating from the center of the bait distribution zone and extended several hundred meters beyond the bait distribution boundary. Traps (range: 62–84, $\bar{X}=73$) were distributed along 6 transects within each web and restricted to forest habitat along transects. The length of individual transects averaged ~914m but ranged from 375 to 1,275 m based on the shape of forest patches and distribution of agricultural fields within study cells. Thus, the effective area trapped during post-baiting sampling covered a substantially larger area than our initial trapping prior to bait distribution in an effort to capture individuals beyond bait distribution boundaries. We attempted to orient transects through forested corridors or other extensive tracts of forest to maximize the length of trap-line transects. However, for forest patches bounded by expansive agricultural fields on one edge this was not feasible and transects on these edges were ended at field edge boundaries.

All raccoons and Virginia opossums captured during post-baiting trapping efforts were processed as outlined above with the exception that we also collected ≥8 whiskers from each individual to evaluate bait uptake [9]. Whiskers were placed in a paper envelope and stored in the dark at room temperature until examination.

### Whisker Analyses

We mounted whiskers from each individual onto one to three slides using Flouromont G (Southern Biotech, Birmingham, AL, USA) oriented with the root tips near the labeled edge of the slide. We examined each slide using 2.5x magnification with a fluorescent microscope comprised of a 100w high-pressure mercury bulb and an RB TRITC filter block (Leica Microsystems, Wetzlar, Germany). A single observer evaluated each slide and classified an individual as marked when we detected a band on ≥1 whisker [43]. Once this initial evaluation was complete a second observer blindly surveyed a random selection of 186 individuals to check for agreement.

### Statistical Analyses

We used logistic regression to model the probability of RB uptake for each species as a function of treatment, age, and sex. Treatment was a categorical variable indicating whether the animal was captured in a treatment or control site. Age was a categorical variable that included juvenile, yearling, or adult for raccoons, and juvenile or adult for opossums. Each animal was considered positive or negative for marking consistent with RB uptake based on the results of whisker analyses as described above. Given the small number of predictor variables, we constructed a set of 17 candidate models that included every combination of variables and their 1^st^ order interactions, as well as an intercept-only model. For each species we assessed goodness of fit of the global model (which included all 1^st^ order interactions) using the le Cessie—van Houwelingen—Copas—Hosmer unweighted sum of squares test for global goodness of fit [44]. We ranked models based on AIC_c_, and assessed relative model performance based on ∆AIC_c_ values, and Akaike weights (*w*_i_, [41]).

### Spatial Analyses

To quantify the extent of the effective area treated, as well as the probability of bait acceptance as a function of distance from bait distribution centers, we estimated kernel density UD’s at each site based on the locations of all traps in which a RB marked raccoon was captured. For RB marked raccoons captured at multiple trapping sites all capture locations were included in the development of UD’s. From the resulting UDs we calculated the 90% and 50% utilization contours, and the area within contour. Ninety percent contours were developed to quantify a conservative extent of the overall effective area treated, eliminating extraneous movements and outliers, whereas 50% contours were used to determine the extent of the primary area treated following bait distributions. All kernel density calculations were performed using the adehabitat package [45] in R [46]. Additionally, for each site we calculated the distance from the edge of the baiting zone to the farthest trap in which an RB marked raccoon was captured.

We used UD estimates at each site to quantify the relative probability of capturing an RB marked raccoon as a function of distance from the center of the baiting zone. To accomplish this, we standardized UD values at each site to range from 0–1 so that cells with the largest value = 1 and the lowest value = 0. This was necessary to make meaningful comparisons across sites. We then extracted the standardized UD value associated with locations of all traps in each web. As traps were placed at 75 m intervals along each transect, we averaged the standardized UD estimates associated with traps occurring at each 75 m interval along transects. This allowed us to calculate the relative decay in UD probability moving away from the center of the trapping grid at each site. As transects and trapping locations were specifically placed in forested habitats in which raccoons were most likely to occur (see post-baiting mark-recapture, above), we estimated probability decay along these transects to avoid biases that would have resulted from sampling UD estimates in habitats that were not sampled and were not likely to contain raccoons. As such, the results should be interpreted as the relative decay in probability of capture of RB marked raccoons in suitable habitat as a function of distance from the center of each bait zone.

## Results

### Initial Mark-Recapture Sampling

During the initial mark-recapture period we captured 217 (123 M, 94 F) raccoons and 56 (26 M, 30 F) Virginia opossums over 4,800 trap nights. The number of unique individuals captured ranged from 14 to 41 ($\bar{X}=30$) for raccoons and 2 to 12 ($\bar{X}=7$) for opossums among sites. The number of competing models (≤2 AIC_c_) resulting from our analysis of capture histories in Program MARK ranged from 1 to 3. Model averaged estimates of raccoon abundance ranged from 18 to 42 ($\bar{X}=31$). Based on estimates derived from our MARK models, estimates of raccoon density ranged from 13 to 30 raccoons/ km^2^ ($\bar{X}=22$ / km^2^) among sites.

### Post-Baiting Mark-Recapture Sampling

We caught 478 (273 males, 205 females) unique raccoons and 108 (47 males, 62 females) opossums over 5,840 trap nights during post-baiting trapping. Of which, 122 (25%) raccoons and 28 (26%) opossums were individuals that had been captured and tagged during initial mark-recapture trapping efforts to estimate density. This increase in total numbers of individuals caught reflects the increase in the effective trapping area during post-baiting trapping due to our effort to capture individuals beyond the trapping grid. Within sites, captures ranged from 24 to 108 ($\bar{X}=60$) for raccoons and 9 to 23($\bar{X}=13$) for opossums. Of these, we collected whiskers from 472 raccoons and 106 opossums.

Of the 186 individuals evaluated by two observers, 174 (94%) were classified as either positive or negative for RB exposure by both individuals. Any discrepancies were classified as negative as a conservative measure of bait acceptance. Overall, 25.8% of raccoons exhibited marking consistent with RB exposure, with a range of 12.2%–38.6% marked raccoons among sites (Table 1). Twenty percent of all opossums exhibited RB marks, ranging from 0%–31.3% among sites (Table 1).

**Table 1:** Site-specific demographic parameters and the percentage of raccoons and Virginia opossums that consumed placebo baits containing Rhodamine B (RB) for both control (C—uniform distribution of baits) and treatment (T—stratified bait distribution based on resource selection information) sites in northcentral Indiana, USA.

**Site**	**Density (raccoons/km^2^)**	**Raccoons captured post treatment**	**% marked with RB**	**Opossums captured post treatment**	**% marked with RB**
C1	30	108	20.5	17	11.8
C2	26	42	12.2	13	30.8
C3	24	58	38.6	10	0.0
C4	13	24	24.1	9	22.2
T1	27	63	27.1	22	19.0
T2	21	60	28.3	10	20.0
T3	19	84	20.0	13	25.0
T4	17	39	35.9	15	31.3

### Statistical Analyses

Results of the le Cessie—van Houwelingen—Copas—Hosmer unweighted sum of squares tests indicated sufficient fit of global models for both raccoons (*P* = 0.51) and opossums (*P* = 0.58). Raccoons and opossums had 2 and 6 models with ∆AIC_c_ values ≤2 respectively (Table 2). The best performing model for each species was the model that included sex as the sole predictor variable (*w_i_* = 0.34 and 0.21 for raccoons and opossums respectively), however, these models had little predictive power; pseudo-*R* ^2^ = 0.009 and 0.035 for raccoons and opossums, respectively. Male raccoons tended to test positive for RB uptake at a greater frequency than females, whereas female opossums tended to test positive with a greater frequency than males, although the low predictive power of these models suggests sex did not play an important role in influencing bait uptake (Table 3). There was little evidence that treatment had any influence of the rate of RB exposure as the only raccoon model containing the variable treatment (treatment + sex) had a weight of only 0.14 and a pseudo-*R* ^2^ of 0.01.

**Table 2:** Highest ranking logistic regression models (∆AIC_c_ ≤ 2) of Rhodamine B uptake by raccoons and opossums on 8 study sites in the Upper Wabash River Basin, Indiana.

**Model**	**K**	**AIC_c_**	**∆AIC_c_**	***w_i_***	**Pseudo R^2^**
Raccoon					
Sex	2	531.89	0.00	0.34	0.009
Treatment + Sex	3	533.67	1.78	0.14	0.010
Opossum					
Sex	2	109.08	0.00	0.21	0.035
Treatment + sex	3	110.22	1.15	0.12	0.049
Treatment*Sex	4	110.23	1.15	0.12	0.080
Treatment	2	110.35	1.28	0.11	0.016
Sex + Age	3	110.81	1.73	0.09	0.041
Age	2	110.96	1.88	0.08	0.007

K = Number of model parameters, AIC_c_ = Aikaike’s Information Criterion adjusted for small sample size, ∆AIC_c_ = Difference in AICc relative to the smallest value, *w_i_* = AICc weight.

**Table 3:** Percent of population marked with Rhodamine B following placebo baiting by age and sex class in control and treatment sites for raccoons and opossums (actual numbers of individuals in parentheses).

	**% Rhodamine B marked (*N*)**		
	**Control**	**Treatment**	**Total**
Raccoon			
Male	27.1 (36)	29.1 (39)	28.1 (75)
Female	20.2 (19)	21.8 (24)	21.2 (43)
Adult	21.8 (31)	28.1 (39)	24.9 (70)
Yearling	25.0 (10)	30.2 (13)	27.7 (23)
Juvenile	31.1 (14)	18.0 (11)	23.6 (25)
Opossum			
Male	4.8 (1)	22.7 (5)	14.0 (6)
Female	25.9 (7)	26.5 (9)	26.2 (16)
Adult	15.0 (6)	24.3 (9)	19.5 (15)
Juvenile	25.0 (2)	26.3 (5)	25.9 (7)

### Spatial Analyses

**Figure 2:**
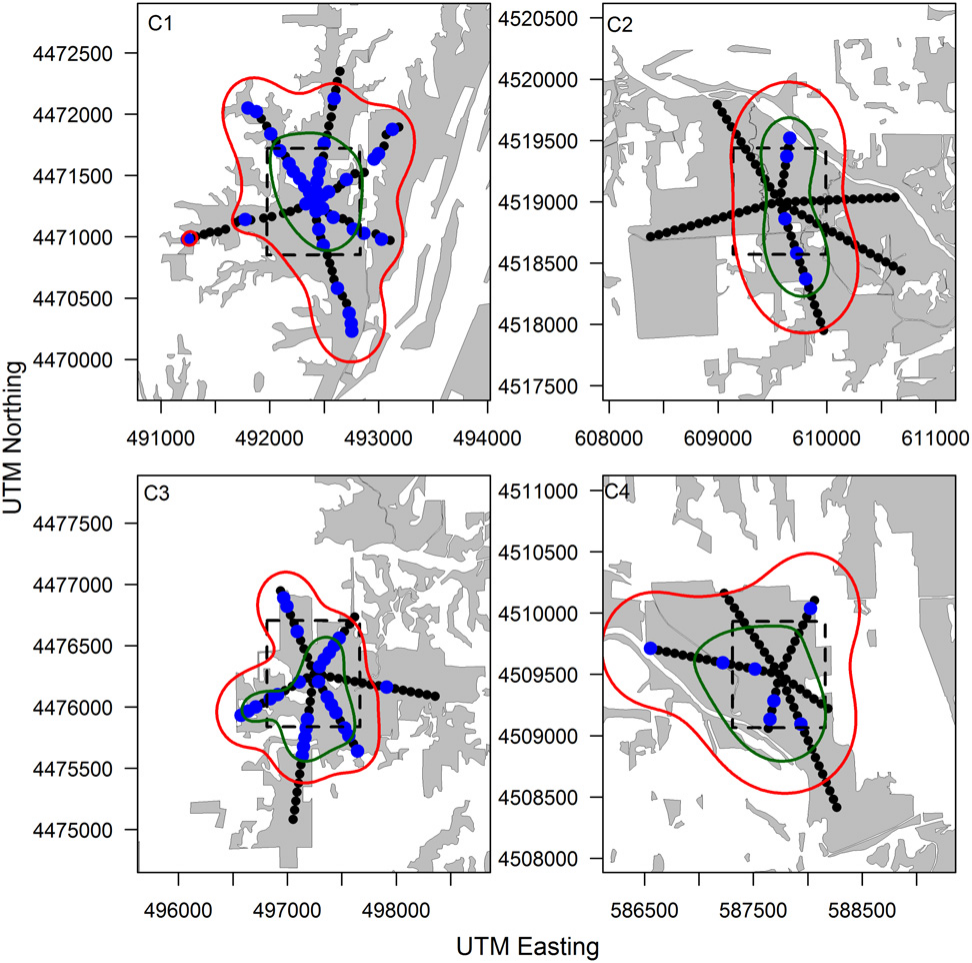
Configuration of control site trapping webs illustrating the distribution of Rhodamine B marked captures (blue), the bait distribution zone (dashed line), and 90% (red) and 50% (green) utilization distributions estimated from the distribution of Rhodamine B marked raccoons. Transects were oriented through forested areas (gray) and did not extend into agricultural areas (white) based on limited use of agricultural interiors by raccoons in our study area. Each panel represents a unique study site where Rhodamine B baits were placed in a uniform distribution within the bait distribution zone (dashed line).

**Figure 3:**
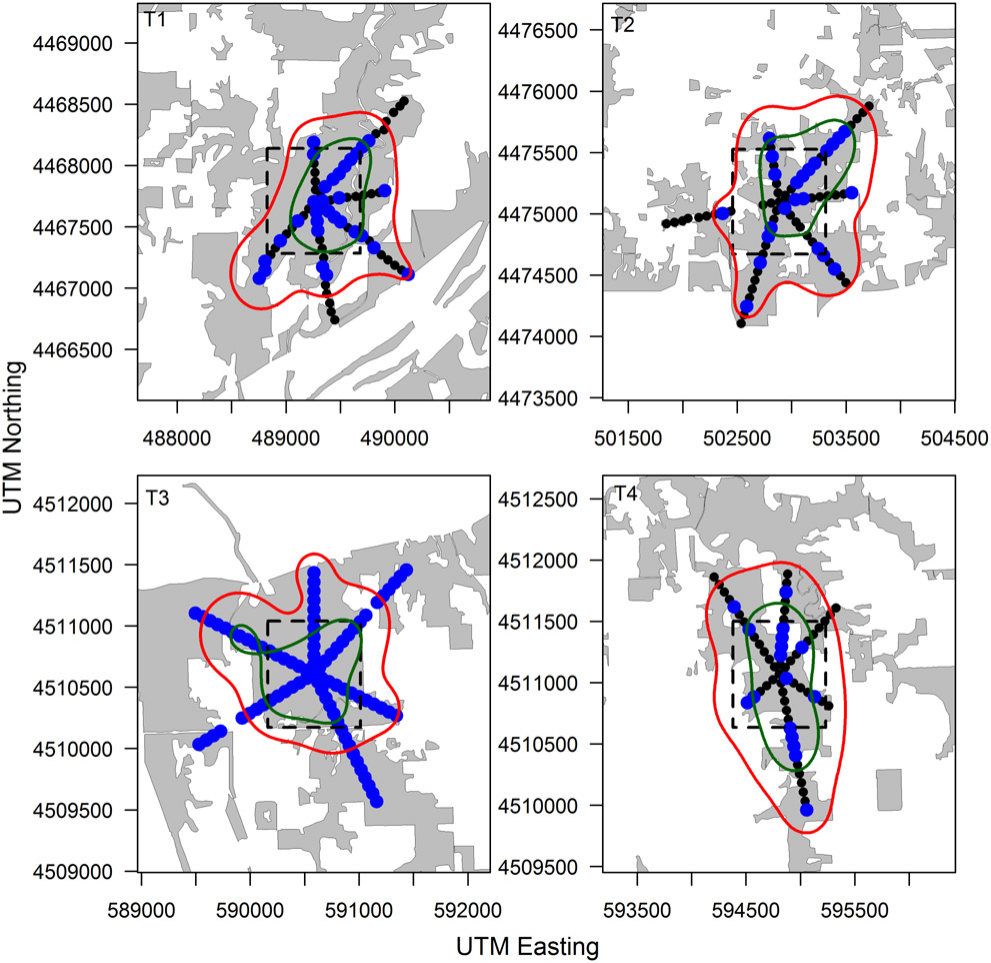
Configuration of treatment site trapping webs illustrating the distribution of Rhodamine B marked captures (blue), the bait distribution zone (dashed line), and 90% (red) and 50% (green) utilization distributions estimated from the distribution of Rhodamine B marked raccoons. Transects were oriented through forested areas (gray) and did not extend into agricultural areas (white) based on limited use of agricultural interiors by raccoons in our study area. Each panel represents a unique study site where Rhodamine B baits were placed in a stratified distribution within the bait distribution zone (dashed line) based on raccoon resource selection patterns in our study area.

RB marked raccoons were captured in traps located up to 753 m from the edge of the bait zone (Table 4). Few RB marked raccoon recaptures were recorded at sites C2 (n = 6) and C4 (n = 7), and as such we recognize that this may have biased kernel density estimates at these sites. At all sites the area of the 90% UD contour was larger than area in which bait was placed (Table 4, Figs. 2 and 3). Ninety percent UD areas ranged from 98.8–240.1 ha greater than bait zone areas (Table 4). Conversely, 50% UD areas were often smaller in area than bait zones (Table 4). Although the small number of sample sites precludes the ability to test for significant differences statistically, there was little apparent difference between control and treatment sites regarding the area over which exposure RB marked raccoons where likely to be captured, or the distance from the baiting zone in which marked raccoons where likely to be captured. Sample sizes of RB marked opossums among sites was too small to construct meaningful UD estimates.

**Table 4:** Number of traps placed, size of the area in which Rhodamine B baits were placed, 50% and 90% kernel utilization density (UD) areas of raccoon recaptures exhibiting Rhodamine B marks, and the distance from the center of the trapping web of the trap in which the furthest RB marked recapture occurred on 8 study sites in the Upper Wabash River Basin, Indiana.

**Site**	**Traps**	**Bait Zone (ha)**	**50% UD (ha)**	**90% UD (ha)**	**Furthest marked capture (m)**
C1	82	74.1	63.7	265.2	731.3
C2	82	74.0	66.1	190.7	201.0
C3	70	74.0	57.2	166.3	245.3
C4	66	74.0	97.1	314.1	753.4
T1	69	73.3	50.8	172.1	475.1
T2	69	73.0	57.3	189.4	425.3
T3	84	74.0	60.2	193.0	396.0
T4	62	73.7	68.4	206.2	673.3

**Figure 4:**
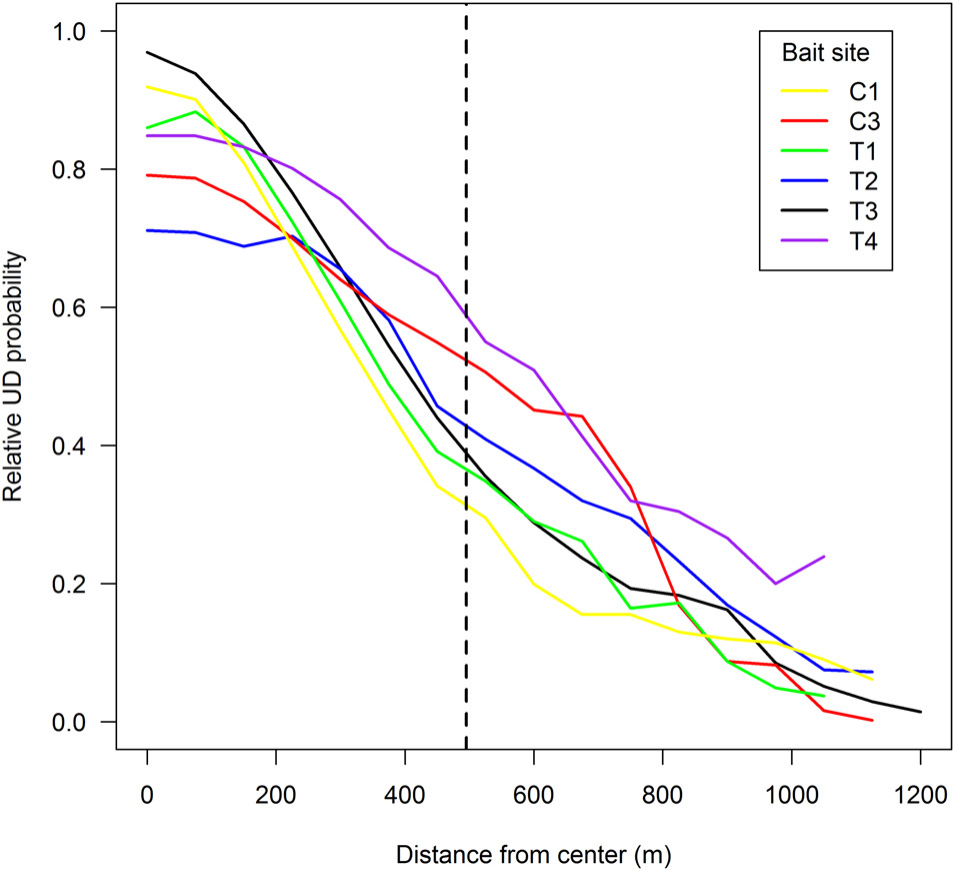
Decay in the relative probability of rabies placebo bait consumption as a function of distance from the center of the bait distribution area. The dashed line represents the approximate edge of the bait zone across the six sites. Two sites were excluded due to low sample sizes.

The pattern of decay in the probability of RB uptake as a function of distance from the center of the baiting zone was similar across sites; the greatest probability occurred at the center of each web and tended to drop quickly as distance from the center increased, even within the bait zone itself (Fig. 4). There was no apparent difference between treatment and control sites. The average distance along transects from the center to the edge of each bait zone was 495 m, and there was no appreciable difference in the shape of curves between traps within the bait zone and those outside. We did not create decay curves for sites C2 and C4 to avoid potential biases resulting from low sample sizes in these sites.

## Discussion

Despite the incorporation of resource selection data into bait distribution models, rates of bait consumption did not differ between treatment and control sites for raccoons in our study. This pattern likely is a reflection of the underlying constraints imposed by the lack of and fragmentation of forested habitat on animal movements, creating hotspots of animal density and activity centered on the distribution of native habitats. As a result, landscape-level processes (i.e. distribution of native habitats) can have a greater influence on general patterns of animal movement behavior in highly fragmented landscapes than the fine-scale distribution of resources within remnant habitat fragments, creating an underlying bias in animal activity that is masked by investigation of resource selection at fine spatial scales [39, 47].

Within fragmented ecosystems animal movements are primarily concentrated within forest interiors, urban parks or residential areas, or along forest-anthropogenic interfaces [35, 36, 47]. As a result, bait acceptance can differ considerably between native and anthropogenic habitats due to the limited use of matrix interiors by raccoons [25, 34, 39]. Therefore, although our data suggest stratified baiting based on fine-scale animal movement patterns is unlikely to improve uptake of vaccine baits at localized spatial scales in fragmented landscapes, incorporation of animal movements into the development of bait distribution strategies likely is critical in fragmented ecosystems at broad spatial scales. Specifically, movement patterns of many mesopredators appear to be most constrained by the distribution of native habitats in highly fragmented landscapes and thus distribution of baits at the level of individual habitat fragments may be the most appropriate scale for maximizing the efficiency of baiting programs in fragmented ecosystems.

The density of baits distributed in this study, based on estimated population sizes within each site (~10 baits/raccoon), was consistent with or higher than those currently used in ORV baiting programs, ranging from 69–162 baits/km^2^ ($\bar{X}=130$; [1]). Despite this, bait acceptance rates generally were low for raccoons, ranging from 12–39% among sites. This is surprising given that this study was conducted during the spring when agricultural food resources are absent. Nonetheless, these data are consistent with, and in some cases lower than, acceptance rates previously reported in the literature, contributing to the growing body of evidence that multiple baiting efforts or increased densities of baits may be needed to achieve population level immunity [26, 48]. The low level of bait acceptance in this and previous bait uptake research in fragmented landscapes (e.g., [9]) is particularly surprising given that raccoon activity is constrained and highly aggregated within forested habitats in fragmented landscapes, likely increasing the potential frequency of bait encounters due to the extensive overlap of baits with animal movement activity. Such low levels of bait acceptance, despite the increased likelihood of bait encounters, suggests factors such as non-target consumption or bait monopolization may play important roles in limiting bait uptake by target species in fragmented areas [9]. Alternatively, bait flavor may play an important role in spatial variability in uptake among study sites, depending upon preference and local availability of similar food items [49].

Subsequent to distribution, baits are rapidly consumed by both target and non-target species, with as many as >99% of baits consumed within 2 weeks and 90% taken within 3 days [11]. Of the baits taken in their study, Smyser et al. [11] reported 95% were consumed by raccoons or opossums, suggesting few other non-target species play an important role in bait competition in agricultural ecosystems supporting high densities of these mesopredators. Given that only 22 opossums consumed ≥1 bait in our study, and in some sites no opossums exhibiting RB marks were captured, opossum consumption of baits likely was not a limiting factor for raccoons. Thus, the limited uptake of baits by raccoons among our study sites suggests that other factors, in addition to bait competition with non-targets, appear to play an important role in limiting uptake by raccoons. In particular, it is unknown what proportion of raccoons or opossums consume multiple baits. Such scenarios could be exasperated in landscapes where baiting occurs in discrete focal areas or along transects spaced sufficiently apart where the probability of an individual encountering a bait may vary depending on the proportion of home range overlap with the bait distribution area or where dominant or more active individuals may dominate bait consumption.

Given that intersexual and age-specific differences in home range size and movement rates have been observed among many mesopredators [36, 50–51], monopolization of baits by more active individuals could contribute to variation in bait encounters among individuals. However, few studies have documented age or sex-specific differences in bait acceptance [18, 26, 48]. In our analyses, the model including only gender received the most support for both raccoon and opossum bait acceptance, although, neither model explained much of the variance (<4%) in bait acceptance. Thus, our study provides further support that neither sex nor age is a limiting factor in bait uptake in free-ranging raccoon populations. Alternatively, the extent of home range overlap with bait distribution areas appears to play a more substantial role in influencing bait acceptance as the probability of bait exposure decreased rapidly with increasing distance from bait distribution centers.

Although many ORV baiting programs distribute baits over large geographic areas, targeted baiting does occur within urban parks and discrete forest patches in heterogeneous landscapes or at small spatial scales in response to localized outbreaks [1, 18, 48]. Similarly, targeted baiting is often used to reduce prevalence of parasites or other diseases at local scales, particularly for programs targeting health of humans or imperiled species. For example, focal-area treatments have been used to reduce human exposure to *Echinococcus multilocularis* in urban areas [52] and similar treatments have been used to reduce *Balisascaris* prevalence in habitats occupied by endangered Allegheny woodrats [10–11]. However, treatment of small focal areas presents unique challenges due to the increased potential for target animals to move between treated and untreated areas. Such effects may be magnified in landscapes supporting high densities of both target and non-target species as baits typically are available for 1–2 days [11] and thus bait uptake is strongly influenced by the proportion of time individuals spend in baited areas. Moreover, localized baiting creates increased edge to interior ratios within treatment areas, potentially reducing the probability of bait uptake for individuals with home ranges along the periphery of bait distribution areas. Under such scenarios, knowledge of the area effectively treated following bait distributions is critical to the implementation of effective baiting strategies.

As predicted, our data confirm that the probability of bait acceptance decreases with increasing distance from the center of the bait zone, even within the zone itself. This pattern of decreasing bait acceptance towards the periphery of the bait zone undoubtedly reflects the proportion of home range overlap with the treated area, with individuals captured towards the center of the zone having a greater probability of encountering baits. Nonetheless, although the probability of bait acceptance was greater for animals captured within the bait zone, numerous animals captured outside the zone had consumed RB baits. It is interesting to note that the decrease in probability of bait acceptance from the center of the bait zone to the extent of the trapping area was relatively linear and did not rapidly decline beyond the bait zone boundary. Moreover, individuals captured up to 753 m beyond the edge of the bait zone had consumed baits, indicating that localized baiting can influence immunity far beyond bait zone boundaries. Indeed, the effective area treated was approximately 3–10x larger than the actual treatment zone itself among the sites sampled in our study. However, levels of bait acceptance rapidly declined below the 70% threshold believed necessary to eradicate disease and thus the proportion of vaccinated individuals may comprise a small minority of the population at increasing distances from baiting interiors. Collectively, these data suggest focal baiting treatments are effective at creating a buffered area of treated individuals around bait zones or bait stations, but that repeated treatments undoubtedly would be needed to achieve sufficient uptake to eradicate disease. In particular, repeated baiting or the distribution of higher densities of baits along habitat or bait zone perimeters may facilitate increased bait acceptance beyond bait zone boundaries by creating additional opportunities for bait encounters in areas where individuals may have limited home range overlap with baited areas.

## Supporting Information

S1 Table Position coordinates (UTM zone 16) for capture locations of all raccoons that tested positive for Rhodamine B uptake. (CSV)

S2 Table Post bait sampling results. The sex (M = male, F = female), age (A = adult, J = juvenile, Y = yearling, see text for details), site (C = control, T = treatment), and Rhodamine B uptake (0 = negative, 1 = positive) for all raccoons and Virginia opossums captured during post bait sampling.
(XLSX)
